# Effects of nutrition motivational intervention in patients affected by type 2 diabetes mellitus: a longitudinal study in Naples, South Italy

**DOI:** 10.1186/s12889-018-6101-6

**Published:** 2018-10-17

**Authors:** Valeria Di Onofrio, Francesca Gallé, Mirella Di Dio, Patrizia Belfiore, Giorgio Liguori

**Affiliations:** 1 Department of Sciences and Technologies, University of Naples “Parthenope”, Business District, Block C4, 80143 Naples, Italy; 2 Department of Movement and Wellbeing Sciences, University of Naples “Parthenope”, Via Medina 40, 80133 Naples, Italy

**Keywords:** Type 2 diabetes, Nutritional motivational intervention, Management of chronic diseases

## Abstract

**Background:**

Type 2 diabetes (T2D) is associated with a prion diminished quality of life, especially due to the severe complications that it implicates. Changing dietary habits is an absolute priority, as well as implementing nutritional motivational programs. The aim of this study was to verify the effectiveness of a nutritional intervention in improving the health of patients affected by T2D.

**Methods:**

A total of 69 patients participated in a nine-months motivational program focused on the principles of the Mediterranean diet, the classes of nutrients, the distribution of the meals during the day and the dietary choices. During regular meetings, the patients were requested to fill out a questionnaire about their dietary habits and behaviours. Clinical and metabolic parameters were also analysed.

**Results:**

At the end of the intervention the number of people who declared that they ate five meals a day (*p* = 0.006) and preferred to have fruit for snack (*p* = 0.004) increased, while there was a reduction in the use of sweeteners and an elimination of the use of fructose (*p* = 0.05). The total daily consumption of kilocalories (kcal) had been reduced and the percentages of carbohydrates, proteins and lipids, after the intervention, follow the guidelines. In relation to this, a significant improvement (*p* < 0.05) was registered in systolic and diastolic pressure, BMI and waist circumference, as well as in glycaemic values (*p* = 0.018).

**Conclusions:**

A nutritional motivational intervention may be useful in improving dietary habits and health status of patients with T2D. We hope that a similar intervention will be applied in Campania and in other Italian regions.

**Trial registration:**

Registration number is ISRCTN11067689; date of registration: 10/09/2018. Retrospectively registered.

**Electronic supplementary material:**

The online version of this article (10.1186/s12889-018-6101-6) contains supplementary material, which is available to authorized users.

## Background

Diabetes is an important public health problem, one of four priority noncommunicable diseases (NCDs) targeted for action by world leaders. Both the number of cases and the prevalence of diabetes have been steadily increasing over the past few decades. The global prevalence (age-standardized) of diabetes has nearly doubled since 1980, rising from 4.7 to 8.5% in the adult population. Separate global estimates of diabetes prevalence for type 1 and type 2 do not exist. The majority of people with diabetes are affected by type 2 diabetes (T2D). The occurrence used to be almost entirely among adults, but now it occurs in children too [1–3].

In recent decades the prevalence of T2D has increased worldwide, with values higher than 5% in Italy [4, 5].

T2DM is associated with a diminished quality of life, especially due to the severe complications that it implicates, leading to possible reductions in life expectancy of up to 10 years [6, 7].

Diabetes prevention and control may be influenced by behavioral factors such as diet and physical activity (PA). A healthful eating pattern is one of the key components of diabetes management [8].

The importance of interventions for Education and Promotion of Health is also emphasised by the Italian National Prevention Plan 2014–2018, which puts diabetes among the priorities of the National Health Service (http://www.salute.gov.it/imgs/C_17_pubblicazioni_2285_allegato.pdf).

Several studies show that motivational nutrition programs and training for patients with T2D can lead to health benefits (weight reduction, decreased cardiovascular risk and improved physical function) especially when these interventions include a cognitive-behavioral approach [9–16].

Many trials report that a balanced diet, body weight maintenance and regular exercise reduce the risk of T2D progression by 30–60% in people with impaired glucose tolerance [17–19].

Salas-Salvadó J et al. assert that the Mediterranean diet improves glucose homeostasis, measurable as a reduction in glycemia and glycosylated hemoglobin, and reduces by 30% the probability of developing T2D [20].

It is a priority to intervene on eating habits, implementing nutritional motivational programs. As also recommended by the guidelines for the treatment and management of diabetes [21], it would be ideal to implement an interdisciplinary intervention to inform people affected by the disease about the principles of a healthy diet and its benefits [22, 23].

In Italy, the use of the group care model, which is based on a clinical-educational approach, seems to be cost-effective and successful in improving diabetes management and quality of life in patients with T2D [24–26]. However, the use of therapeutic education for these patients in clinical practice encounters difficulties mainly due to the lack of facilities destined to this activity, and to the scarcity of trained operators [27].

We performed a study to verify the feasibility and efficacy of a nutritional motivational intervention in improving the health and disease self-management of patients affected by T2D in Naples, South Italy.

## Method

This study is part of a health promotion intervention sponsored by the National Center for Prevention and Control of Diseases of the Italian Ministry of Health. It aimed to evaluate possible changes in food habits and health perception in a sample of individuals with T2D before and after a 9 month nutritional motivational program. Anthropometric parameters (BMI, waist measurement, hip measurement) and endocrinal-metabolic data (blood pressure, heart rate, glycaemia, HbA1c, total cholesterol, HDL, LDL, triglycerides, creatinine) were assessed before and after the intervention. Dietary habits were also evaluated. The investigation wasn’t experimental, therefore a trial registration was not provided.

### Participants and setting

In the preliminary phase of the study (3 months), physicians and diabeticians identified patients who were eligible and invited them to participate in the investigation. Eligibility criteria were being between 50 and 70 years old, living in the community, having been diagnosed with T2D at least 1 year prior, absence of major complications of diabetes. All participants were informed about the purpose of the study and the use of resulting data, and signed an informed consent for being included in the intervention. Participants joined the study groups on a voluntary basis.

Individuals who decided to take part in the nutritional motivational program formed the intervention group (IG) while recruited patients who decided not to follow the program were included in the control group (CG).

### Intervention

The intervention lasted 9 months. The nutritional program was structured in quarterly group meetings conducted by a trained nutritionist, who discussed with patients the role of diet in diabetes control, Mediterranean diet benefits, healthy food choices, and how to manage their own nutrition through an adequate daily distribution of meals, using photo books containing examples of meals, as well as to learn a correct interpretation of food labels. Detailed information about how to prevent and manage hypoglycemia was given.

All procedures followed were in accordance with the ethical standards of the Responsible Committee on Human Experimentation (institutional and national) and with the Helsinki Declaration of 1975, as revised in 2008. Anonymity of personal data was guaranteed. All participants signed an informed consent form. For ethical reasons, all the participants in the control group received information about the principles of a healthy diet from their physicians. The study protocol was approved by the National Centre for the Prevention and Control of Disease (CCM).

#### Outcomes

This intervention aimed to promote the well-being of patients, involving and making them aware of their dietary choices. During the program some tools were provided to control body weight and maintain glucose homeostasis.

Dietary habits and behaviours, anthropometric and endocrinal-metabolic parameters, were assessed before (t0) and at the end (t3) of the activities in participants to study the effects of the intervention.

During the periodical meetings, a questionnaire of 29 questions on dietary habits and behaviours previously validated in another intervention granted by the National Center for Prevention and Control of Diseases of the Italian Ministry of Health [28] was carried out (see Additional file 1). This tool enabled the analysis of the daily consumption and type of foods eaten at breakfast, or as snacks, at lunch and dinner.

The average daily amount, average daily calories consumed, distribution into micro and macro nutrients and caloric breakdown between meals during the day were assessed and compared for each patient according to the dietary anamnesis software WinFood7® (Medimatica S.u.r.l. – Teramo, Italy).

### Statistical analysis

The answers to the questionnaires on dietary habits and behaviours of the IG and the CG, at the start and the end of the program, were analysed using the χ^2^ test.

The Student t test and variance analysis (ANOVA) were applied for the comparison of endocrinal-metabolic parameters (and their variations) between the IG and the CG at the start (t0) and end (t3) of the intervention. A *p* value of 0.05 was considered as the level of significance. All the analyses were carried out using IBM SPSS statistics version 23 for Windows (SPSS, Chicago, IL, USA).

## Results

### Participation and socio-demographic characteristics of the participants

Out of the 213 subjects who accepted to participate in the study, 69 (32.4%) completed the program (IG = 47 M and 22F; mean age 64 ± 5.57) and 210 controls who participated in the follow-up (CG = 108 M and 102 F; mean age 65 ± 7.46) were included in the final analysis.

Table 1 shows the socio-demographic characteristics of the two groups.

**Table 1:** Socio-demographic characteristics of the Intervention Group (IG) and of the Control Group (CG)

Variables	IG		CG	
	N.	%	N.	%
Age	64 ± 5.57		65 ± 7.46	
Gender				
Males	47	68.1	108	51.4
Females	22	31.9	102	48.6
Educational qualifications				
None	1	1.5	16	7.6
Primary school	16	23.2	64	30.5
Middle school	22	31.9	24	11.4
High school diploma	23	33.3	72	34.3
University degree	5	7.2	24	11.4
No answer	2	2.9	10	4.8
Occupation				
Unemployed	3	4.3	6	2.8
Housewife	18	26.1	54	25.7
Pensioner	29	42.0	34	16.2
Tradesman	5	7.2	24	11.4
Office worker	9	13.1	72	34.3
Self-employed	4	5.8	10	4.8
No answer	1	1.5	10	4.8

### Questionnaire on dietary habits and behaviours

Table 2 shows the answers with statistically significant differences between t0 and t3 in IG patients; no statistical relevance was found among the CG responses (an additional file is more detailed (see Additional file 1).

**Table 2:** Main dietary habits and behaviours of the Intervention Group (IG) and the Control Group (CG) at t0 and t3

	IG					CG				
Questions	t0		t3		χ2 (p)	t0		t3		χ2 (p)
	N.	%	N.	%		N.	%	N	%	
What do you use to sweeten food and drinks?										
White sugar	25	36.2	23	33.3	0.05	73	34.8	68	32.4	0.85
Brown sugar	7	10.2	13	18.8		18	8.5	21	10.0	
Fructose	7	10.2	0	–		21	10.0	17	8.1	
Miele	0	–	2	2.9		3	1.4	5	2.4	
Sweeteners	13	18.8	12	17.4		41	19.6	48	22.8	
Nothing	17	24.6	19	27.6		54	25.7	51	24.3	
Do you eat five meals a day?										
Yes	30	46.4	46	66.7	0.006	91	42.3	100	47.6	0.38
No	39	53.6	23	33.3		119	57.7	110	52.4	
Do you snack on fruit?										
Yes	16	23.2	32	46.4	0.004	71	33.8	78	37.1	0.48
No	53	76.8	37	53.6		139	66.2	132	62.9	
Do you regularly eat fish?										
Yes	50	72.4	63	91.3	0.004	188	89.5	191	90.9	0.62
No	19	27.6	6	8.7		22	10.5	19	9.1	
What do you usually order at a coffee shop?										
Bitter orangeade	26	37.8	17	24.6	0.02	71	33.8	75	35.7	0.95
Freshly squeezed orange juice without sugar	29	42.0	41	59.4		115	54.8	112	53.3	
Ice lolly	0	–	4	5.8		18	8.6	16	7.7	
Ice cream cone	14	20.2	7	10.2		6	2.8	7	3.3	

In particular, there was a reduction in the consumption of white sugar and an increase of brown sugar; the fructose was eliminated (*p* = 0.05) and the consumption of five meals a day became deeply rooted (*p* = 0.006). Having fruit for snack doubled (*p* = 0.004) and there was an increase in the regular consumption of fish (p = 0.004).

The ice cream consumption was halved and the patients preferred low-calorie drinks such as freshly-squeezed orange and ice lollies (*p* = 0.02).

The outcomes show that the habit of having breakfast, already high at t0 (95.7%), improved significantly (100%) (*p* = 0.08). The seasonal fruits and vegetables consumption increased (*p* = 1.48), while meat consumption decreased (*p* = 0.32), as that of cured meats, cheeses and bakery products containing oil and lard; there was a reduction in simple sugars intake in the form of sweets and candies and also in alcohol consumption outside the meal (*p* = 1.82) (data not show).

The results of WinFood7® related to the dietary consumption of the IG are shown, at t0 and t3, in Table 3.

**Table 3:** Results of WinFood7® at t0 and t3 in relation to the food consumption of the IG

		Average (t0)	Average (t3)
LIST OF COMPONENTS			
Calories	Kcal	2152.88	1588.04
Alcohol	Kcal	103.18	92.40
Protein	g	119.07	73.22
Lipids	g	100.83	49.83
Glycids available	g	176.94	199.83
Starch	g	48.15	67.61
Oligosaccharides	g	90.91	92.71
Total fibre	g	20.78	21.84
Cholesterol	mg	954.21	359.70
Saturated fatty acids	g	34.17	15.61
Polyunsaturated fatty acids	g	9.35	5.23
Monounsaturated fatty acids	g	49.02	25.58
Calcium	mg	1379.68	897.76
Sodium	mg	2794.45	1252.95
Potassium	mg	4207.25	3872.02
% BREAKDOWN AMONG MEALS (calories)			
Breakfast	%	29.40	18.26
Snacks	%	22.08	18.12
Lunch	%	29.84	36.86
Dinner	%	18.68	26.77

The total kcal were reduced, while the fibre intake stayed almost identical, below the recommended level [21]. The macronutrients distribution, carbohydrates percentages, protein and fats at t3 comply with guidelines. The relationship between starch and simple carbohydrates (in favour of the latter at t0) is more balanced at t3, but not optimal; there was a considerable reduction of saturated fats.

The calories distribution between meals after 9 months is closer to that recommended for a balanced diet.

There were no significant changes between t0 and t3 in the CG (data not show).

### Anthropometric and endocrinal-metabolic parameters

After 9 months, the anthropometric and endocrinal-metabolic parameters in the IG had all improved. The parameters of this group are always better than those found in the CG (Table 4).

**Table 4:** Intra-group and inter-group comparison of clinical-metabolic parameters at t0 and t3 with relative *p* values

	IG					CG					ANOVA
	t_0_		t_3_		t Student	t_0_		t_3_		t Student	
	average	σ	average	σ	P	average	σ	average	σ	P	P
BMI	31.26	9.47	26.85	2.74	0.01*	33.35	11.14	30.57	4.23	0.3	0.045*
Waist measurement (cm)											
Women	100.33	14.03	94.00	17.04	0.49	109.2	7.46	105.2	5.26	0.35	0.33
Men	106.46	11.94	90.37	10.70	0.00*	94.33	16.74	92.16	11.66	0.78	0.03*
Systolic pressure	133.86	13.35	125	13.8	0.04*	136.42	11.99	133.07	10.31	0.44	0.001*
Diastolic pressure	77.5	9.4	71	6.2	0.03*	75.71	9.3	76.53	6.2	0.79	0.001*
Glycaemia (mg/dl)	145.2	39.94	119.23	19.2	0.018*	150.37	80.86	136	23.16	0.49	0.007*
HbA1c(%)	6.6	0.87	6.2	0.68	0.22	7.02	1.81	14.12	19.16	0.12	0.00*
Tot. chol. (mg/dl)	176.26	41.91	177.34	24.71	0.9	185	44.34	181	42.23	0.79	0.00*
HDL (mg/dl)	49	9.2	52	12	0.5	52.11	11.37	49.33	11.11	0.48	0.00*
Triglycerides (mg/dl)	172.34	78	131.86	66.9	0.1	142.6	62.05	155.46	85.18	0.62	0.5
Creatinine (mg/dl)	6.2	23	2.6	2.58	0.57	3.23	2.8	3.35	2.99	0.91	0.3

At the time of enrolment, the members of both groups presented an average BMI which placed them in the primary obesity band (IG 31.26 ± 9.47; CG 33.35 ± 11.4). At the end of the program, the average BMI in both groups seems generally reduced, only those in the IG fall within the overweight band (26.85 ± 2.74; *p* = 0.01).

The average waist measurement at t0, in men and women in the IG and the CG, was above the threshold values set by the WHO (80 cm for women and 94 cm for men). At t3, these average values are reduced in both sexes and in both groups, but only the men in the IG fall within the values recommended by the WHO (*p* = 0.00).

In the CG there were no significant differences in endocrinal-metabolic parameter between the start and the end of the program, unlike the IG (*p* ≤ 0.05 for BMI, glycaemia, waist measurement, systolic and diastolic pressure). The ANOVA test results show significant differences in parameters between the IG and CG.

## Discussion

The increasing prevalence of T2D makes necessary and urgent to intervene on people’s lifestyles, focusing mainly on a balanced diet and movement [29, 30]. Many studies suggest that eating a Mediterranean diet coincides with a reduced incidence of T2D [31–33].

The nutritional education that aims to correct and conscious food choices, associated with the active lifestyle promotion, can provide a renewed and complete therapeutic approach. It is able to offer patients highly effective tools to control the disease and improve the quality of life.

The main findings of this study are that a nine-month community-based nutritional motivational program improves dietary habits and behaviours, anthropometric and endocrinal-metabolic parameters in middle-aged and older subjects with T2D compared to control diabetic subjects undergoing only training about healthy style controlled by physicians and diabeticians. Although they did not join the program randomly but on a voluntary basis, two samples came out from the same population and were similar in age and sex.

The better received messages:

- ✓ the fructose has a great hyper-triglyceridemising power. It was completely eliminated from the diet at the end of the program;
- ✓ it is necessary to eat 5 meals a day to maintain glycidic homeostasis;
- ✓ it is necessary to abandon sugary drinks, seriously implicated in the dynamics of the disease, as reported by various studies [34–36].

Participants improved the ratio of simple vs complex carbohydrates and the content of fibre in their diet, despite not yet complying with optimal values.

A considerable reduction in daily caloric consumption was also found, with a total 50% reduction in levels of cholesterol and fatty acids at t0, with remarkable effects, similar to those related to carbohydrates, on the progression of disease and appearance of complications [37, 38].

The reduced intake of fatty acids in favour of unsaturated fatty acids, after 9 months, is related to the reduced consumption of meat, especially processed products, indicated in the questionnaire [39], and to the increased consumption of fish.

Over the 9 months, the average BMI in the IG passed from level one obesity to overweight values. This parameter, which did not change in the CG, seems to be the most significant indication of the success of the intervention.

Another important result is the waist measurement index found at t3 in the women in the IG which, despite being diminished, does not fall within the thresholds indicated by the WHO. We should remember that the mean age of participants, almost all of whom were post-menopausal, implies that the distribution of body fat is altered, and that it deposits mainly in the abdominal area. Another reason for these results is that basal metabolic values are lower in women than in men, and this makes more difficult to lose abdominal fat.

The relationships with the professionals and their communication skills, as well as the organization and the contents of the sessions were generally appreciated by the participants.

The main limitations of this study are related to the participation of patients. In particular, the poor adherence to the study reflects a lack of acceptance of similar interventions to promote healthy lifestyle in the population considered. Furthermore, the considerable number of dropouts and withdrawals observed among study participants should be considered. Although we have not thoroughly investigated the reasons for dropouts, this could be related to the type of supportive patients received from the staff of these facilities. However, the levels of neglect recorded in this study are comparable to those reported by other similar studies [40–42].

The lack of randomization is another important limit: one would expect that those who were not willing to participate in the motivational program were less motivated to improve their health. However, the improvement of most parameters reported by the controls suggest that the effect of this possible distortion was minimal.

Finally, it should be noted that our intervention involved only individuals aged 50 to 70, while in Italy the highest age group affected by diabetes is that of the elderly aged ≥75 (prevalence rate 19.8%) [5].

In conclusion, a community-based long-term intervention that includes a supervised food motivational program can improve the health of individuals with T2D.

The main criticality found was the significant loss of participants at the follow-up; as already mentioned, only 36.3% of those who started out completed the whole nine-month program.

The reasons for such an important drop-out can be attributed to a lack of commitment at the time of enrolment and of motivation of doctors, insufficient support from expert educators or logistic difficulties, due to family/personal problems which are not always easy to define and solve.

Changes in the anthropomorphic and endocrinal-metabolic parameters found among participants in the activities highlighted the effectiveness of the intervention described, configuring it as a valid form of tertiary prevention transferrable at territorial level.

## Conclusions

The structured intervention turned out to be feasible in the Neapolitan community: it was appreciated by participants and did not require great amounts of personnel or economic resources. As reported in a previous economic analysis, an exercise-based intervention for diabetic subjects could represent a cost-saving strategy for diabetes management, especially for complicated cases [43]. Therefore, the proposed intervention could be considered as an effective and inexpensive tool to improve health and prevent complications in subjects with T2D.

The active involvement and interconnection between the various figures involved (doctors, educators) is decisive in accompanying and actively sustaining patients in the adoption of a more active and healthy life style; this is an element on which it is necessary to insist because a lack of compliance turned out to be the most significant criticality.

Training in health education aimed at healthcare professionals could help reduce the drop-out.

It is evident that the intervention cannot be implemented without a network of collaborators where various components operate synergistically and with equal efficiency, with a view to achieving shared aims.

The involvement of institutions, able to give priority to public health, is the strength of the project. it aims to underline the prevention importance and health promotion in improving the quality of life of diabetic patients.

The patient’s care must go through the remodulation of the ATDP (Assistance-treatment-diagnostic protocol) that includes appropriate interventions for promotion and education in health, through correct dietary choices and an active life.

Considering the positive aspects of the project, we hope that this model, with the appropriate improvements, can continue to be applied and extended to other areas and to the management of other chronic situations.

## Additional file

Additional file 1: Questionnaire on dietary habits and behaviours. Questionnaire of 29 questions on dietary habits and behaviours previously validated in another intervention granted by the National Center for Prevention and Control of Diseases of the Italian Ministry of Health. (DOC 114 kb)

## Abbreviations

BMI: Body Mass Index
CCM: National Centre for the Prevention and Control of Disease
CG: Control group
GP: General Practitioners
HbA1c: Glycated haemoglobin
HDL: High-Density Lipoprotein
IG: Intervention group
LDL: Low-density Lipoprotein
T2D: Type 2 diabetes
WHO: World Health Organization
