# Supplementary material for: Effects of nutrition motivational intervention in patients affected by type 2 diabetes mellitus: a longitudinal study in Naples, South Italy
Source: BMC Public Health. 2018 Oct 17;18:1181. doi: 10.1186/s12889-018-6101-6 (PMC6192365; doi:10.1186/s12889-018-6101-6)
Supplement: Supplementary file 1 — Questionnaire on dietary habits and behaviours. Questionnaire of 29 questions on dietary habits and behaviours previously validated in another intervention granted by the National Center for Prevention and Control of Diseases of the Italian Ministry of Health. (DOC 114 kb) [file 12889_2018_6101_MOESM1_ESM.doc]

**Questionnaire on dietary habits and behaviours**

*Dear Sir/Madam*

*Some habits or situations that are part of everyday life are listed below. Please choose the answer that best suits you, indicating it with a cross.*

1. Do you have **breakfast** every morning? ⁪ Yes ⁪ No
2. What do you drink at **breakfast**?

Choose one of the values indicated: **1** = Fair amount **2** = Average amount **3** = Large amount

|  | **Amount** |
| --- | --- |
| Milk |  |
| Coffee |  |
| Tea |  |
| Ginseng |  |
| Barley |  |
| Alcohol |  |
| Water |  |
| Other |  |

1. What do you eat at **breakfast**?

Choose one of the values indicated: **1** = Fair amount **2** = Average amount **3** = Large amount

|  | **Amount** |
| --- | --- |
| Croissants, doughnuts, brioches, etc. |  |
| Rusks / French Toast |  |
| Bread |  |
| Biscuits |  |
| Pre-packed snacks |  |
| Fruit |  |
| Cured meats |  |
| Cheese |  |
| Eggs |  |
| Pizza or other carbohydrates |  |

1. Where and how do you have **breakfast**?

|  At home, alone, standing up |
| --- |
|  At home, alone, sitting at the table |
|  At home, in company, standing up |
|  At home, with family, sitting down |
|  In a public place, alone, standing up |
|  In a public place, alone, sitting down |
|  In a public place, in company, standing up |
|  In a public place, in company, sitting down |

1. If you don’t have breakfast, explain why: __________________________________________________

_________________________________________________________________________________

1. Do you eat one or more **snacks** during the day? ⁪ Yes ⁪ No
2. What do you drink with a **snack**?

Choose one of the values indicated: **1** = Fair amount **2** = Average amount **3** = Large amount

|  | **Amount** |
| --- | --- |
| Milk |  |
| Coffee |  |
| Tea |  |
| Ginseng |  |
| Barley |  |
| Alcohol |  |
| Water |  |
| Other |  |

1. What do you eat as a **snack**?

Choose one of the values indicated: **1** = Fair amount **2** = Average amount **3** = Large amount

|  | **Amount** |
| --- | --- |
| Croissants, doughnuts, brioches, etc. |  |
| Rusks / French Toast |  |
| Bread |  |
| Biscuits |  |
| Pre-packed snacks |  |
| Fruit |  |
| Cured meats |  |
| Cheese |  |
| Eggs |  |
| Pizza or other carbohydrates |  |

1. Where and how do you eat your **snack**?

|  At home, alone, standing up |
| --- |
|  At home, alone, sitting at the table |
|  At home, with family, standing up |
|  At home, with family, sitting down |
|  In a public place, alone, standing up |
|  In a public place, alone, sitting down |
|  In a public place, in company, standing up |
|  In a public place, in company, sitting down |

1. Do you usually have **lunch**? ⁪ Yes ⁪ No
2. What do you drink at **lunch**?

Choose one of the values indicated: **1** = Fair amount **2** = Average amount **3** = Large amount

|  | **Amount** |
| --- | --- |
| Sugary fizzy drinks |  |
| Coffee |  |
| Tea |  |
| Wine |  |
| Barley |  |
| Beer |  |
| Water |  |
| Other |  |

1. What do you drink at **lunch**?

Choose one of the values indicated: **1** = Fair amount **2** = Average amount **3** = Large amount

| **A typical LUNCH…** | **Amount** |
| --- | --- |
| Starter |  |
| First course – Simple Carbohydrates |  |
| First course – Pulses |  |
| First course – Elaborate Carbohydrates |  |
| Meat |  |
| Fish |  |
| Vegetables |  |
| Salad |  |
| Eggs |  |
| Fruit |  |
| Dessert |  |

1. Where and how do you have **lunch**?

|  At home, alone, standing up |
| --- |
|  At home, alone, sitting at the table |
|  At home, with family, standing up |
|  At home, with family, sitting down |
|  In a public place, alone, standing up |
|  In a public place, alone, sitting down |
|  In a public place, in company, standing up |
|  In a public place, in company, sitting down |

1. Do you usually have **dinner**? ⁪ Yes ⁪ No
2. What do you drink at **dinner**?

Choose one of the values indicated: **1** = Fair amount **2** = Average amount **3** = Large amount

|  | **Amount** |
| --- | --- |
| Sugary fizzy drinks |  |
| Coffee |  |
| Tea |  |
| Wine |  |
| Milk |  |
| Beer |  |
| Water |  |
| Other |  |

1. What do you drink at **dinner**?

Choose one of the values indicated: **1** = Fair amount **2** = Average amount **3** = Large amount

|  | **Amount** |
| --- | --- |
| Starter |  |
| First course – Simple Carbohydrates |  |
| First course – Pulses |  |
| First course – Elaborate Carbohydrates |  |
| Meat |  |
| Fish |  |
| Vegetables |  |
| Salad |  |
| Eggs |  |
| Fruit |  |
| Dessert |  |

1. Where and how do you have breakfast?

|  At home, alone, standing up |
| --- |
|  At home, alone, sitting at the table |
|  At home, with family, standing up |
|  At home, with family, sitting down |
|  In a public place, alone, standing up |
|  In a public place, alone, sitting down |
|  In a public place, in company, standing up |
|  In a public place, in company, sitting down |

1. Do you think you chew your food properly?

 Yes  No  Sometimes

1. Do you ever lose control when eating (e.g.: eating too much food, of different types, very quickly)?  Yes  No
2. If the answer is yes, can you indicate how often this happens and what you eat?

________________________________________________________________________________________________________________________________________________________________________

1. How many meals do you eat during the day?

 1  2  3  4  5

1. Do you usually snack on fruit (at least 4-5 times a week)?

 Yes  No

1. What do you use to sweeten food and drinks?

 White sugar  Brown sugar  fructose  honey  sweeteners

1. Do you usually eat meat (more than three times a week)?  Yes  No
2. Do you usually eat fish (more than three times a week)?  Yes  No
3. You are away from home in the afternoon and a friend invites you to have something to eat or drink in a bar. What do you choose?

 bitter orangeade  unsweetened freshly-squeezed orange juice  ice lolly  ice cream cone

1. When you do food expenditure, what do you buy to eat?

 butter  cured meats and cheeses  bread and breadsticks  beans and peas

1. One afternoon, you go to visit a friend who offers you something to eat or drink. What do you choose?

 sweets  a glass of wine  coffee or tea with sweetener  a digestive drink

1. The fruit you usually eat is:

 grapes  seasonal fruit  apple  I don’t eat fruit

***Thank you for your cooperation***

Place and date, _____________________ signature
